# *TaNRT1.1B*-mediated nitrogen acquisition contributes to salt adaptation and yield-trait maintenance in wheat

**DOI:** 10.1016/j.abiote.2026.100090

**Published:** 2026-09-12

**Authors:** Shuxuan Quan, Yitian Li, Cheng Peng, Qian Zhang, Zhen Wang, Jie Zang, Guiluan Wang, Xian Sheng Zhang, Yifeng Hou

**Affiliations:** a State Key Laboratory of Wheat Improvement, Peking University Institute of Advanced Agricultural Sciences, Shandong Laboratory of Advanced Agricultural Sciences, Weifang, Shandong, 261325, China; b State Key Laboratory of Wheat Improvement, College of Life Sciences, Shandong Agricultural University, Tai'an, Shandong, 271018, China; c State Key Laboratory of Wheat Improvement, College of Agronomy, Shandong Agricultural University, Tai'an, Shandong, 271018, China

**Keywords:** Wheat, Salt tolerance, *TaNRT1.1B*, Nitrogen acquisition, Grain yield traits

## Abstract

Soil salinity is a major environmental stress that limits global wheat production, in part by impairing nitrogen acquisition, which integrates essential nutrient supply with stress-adaptation signaling. Although nitrogen fertilization can alleviate salt stress, the underlying molecular mechanisms remain largely unknown. Here, we show that salt stress suppresses nitrate accumulation in wheat seedlings, as well as reducing root and shoot growth. Comparative transcriptome profiling reveals that nitrate-associated genes and transcriptional regulators are significantly enriched among salt-responsive genes. To explore the functional relevance of this enrichment, we focused on *TaNRT1.1B*, a prominently downregulated nitrate transporter gene, demonstrating its role as a key regulator linking nitrate acquisition and salt-stress responses. Disruption of *TaNRT1.1B* reduced nitrate uptake, impaired ABA responses, compromised salt tolerance, and significantly reduced the values of yield-related traits under salt stress. Supporting the functional relevance of *TaNRT1.1B*, salt-tolerant wheat accessions had significantly higher *TaNRT1.1B* expression and nitrate levels under saline conditions than salt-sensitive lines. Collectively, our results demonstrate that *TaNRT1.1B* links nitrate uptake with salt-stress tolerance to coordinate growth and stress resilience in wheat, providing a potential genetic target for breeding of varieties with improved salt tolerance, nitrogen use efficiency, and grain yield.

## 1 Introduction

Wheat (*Triticum aestivum*) is a staple crop cultivated worldwide, whose stable production is fundamental to the global food security of a growing population [[1], [2], [3]]. However, wheat growth and productivity are constrained by multiple environmental factors. Soil salinity is one of the most detrimental, severely limiting plant growth and threatening wheat production [4,5]. The situation is exacerbated by the continued expansion of saline soils worldwide, driven by climate change, poor irrigation practices, and industrial pollution. This poses a particular challenge for wheat, which—despite being cultivated across a wider geographic range than any other crop—is relatively sensitive to salinity [1,2,4]. Dissecting the molecular mechanisms that underlie salt tolerance in wheat is therefore essential for understanding plant adaptation and sustaining grain production in an era of expanding saline soils.

From a plant physiological perspective, the detrimental effects of salinity manifest as hyperosmotic stress, ion toxicity, and oxidative damage triggered by excessive ROS accumulation [6]. Salt tolerance in wheat is a complex quantitative trait governed by a network of genes involved in ion homeostasis, ROS scavenging, osmotic adjustment, and signal transduction. These functional classes of genes work in concert to maintain ionic balance, reduce ROS levels, transduce stress signals, and modulate cellular metabolism. Characterizing the expression patterns and functional interactions among these genes provides a foundation for improving salt tolerance in wheat through molecular breeding and genetic engineering [[7], [8], [9], [10], [11], [12]].

High concentrations of sodium ions (Na^+^) impair the uptake of water and essential nutrients such as potassium (K^+^), nitrogen, phosphorus, and calcium, leading to ionic imbalance, oxidative damage, and ultimately reduced grain yield and quality [13]. Nitrogen in particular has emerged as a key player in stress adaptation owing to its dual role as an essential macronutrient and a signaling molecule [14]. Nitrogen supplementation has been shown to enhance salt tolerance by regulating osmotic potential, mitigating oxidative damage, maintaining ion homeostasis, stabilizing membrane integrity, and optimizing photosynthetic performance [13,[15], [16], [17]]. Moreover, an increased content of amino acids, direct products of nitrogen assimilation, can enhance both grain yield and salt tolerance in rice [18]. However, less is known about how nitrogen metabolism interacts with salt-stress responses in wheat.

Plants acquire nitrogen primarily in the form of nitrate (NO_3_^−^), whose uptake and assimilation pathways have been extensively characterized in model species. In *Arabidopsis*, the nitrate transporter Nitrate Transporter 1.1 (NRT1.1) functions as both a transporter and a sensor, regulating nitrate-responsive gene expression [[19], [20], [21]]. Upon nitrate perception, NRT1.1 triggers a signaling cascade that leads to the nuclear accumulation of Nodule Inception (NIN)-like protein 7 (NLP7), a master transcription factor that activates nitrate-responsive genes [14,22,23]. In rice, OsNRT1.1B plays a central role in nitrate assimilation and plant growth and also functions as an abscisic acid (ABA) receptor, integrating nitrate and stress signaling pathways [24]. Despite these findings in model species, the potential role of TaNRT1.1 in linking nitrogen metabolism to salt-stress responses in wheat remains to be characterized.

In this study, we sought to characterize the molecular mechanisms by which salt stress affects nitrogen metabolism in wheat. We performed a time-series transcriptome analysis of roots and shoots under salt treatment, integrating morphological and transcriptomic data to identify key regulators that link the nitrogen and salt response pathways. This approach led to the identification of *TaNRT1.1B* as a candidate for the integration of these processes. By combining genetic, physiological, and molecular analyses, we demonstrated that *TaNRT1.1B* functions in coordinating nitrate uptake, ABA responses, stress tolerance, and plant growth in response to salt and nitrogen cues. These findings reveal a functional coupling between nitrate acquisition and salt tolerance mediated by *TaNRT1.1B*, highlighting a molecular entry point for coordination of stress resilience and nitrogen use efficiency (NUE) in wheat.

## 2 Results

### 2.1 Salt stress inhibits wheat seedling growth and reduces nitrate content

To investigate the effects of salt stress on wheat seedlings, we treated plants at the two-leaf- one-heart stage with 100 mM NaCl for various durations (0, 0.5, 3, 12, 24, 48, 72, or 96 h). No significant morphological changes were observed within the first 48 h of treatment. However, after 96 h of salt exposure, both root length and root biomass were significantly reduced compared with those of control plants (Fig. 1A–C). Similarly, shoot height and shoot biomass decreased under prolonged salt treatment (Fig. S1A–C). These results confirm that extended salt stress inhibits the growth of wheat seedlings.

**Fig. 1:**
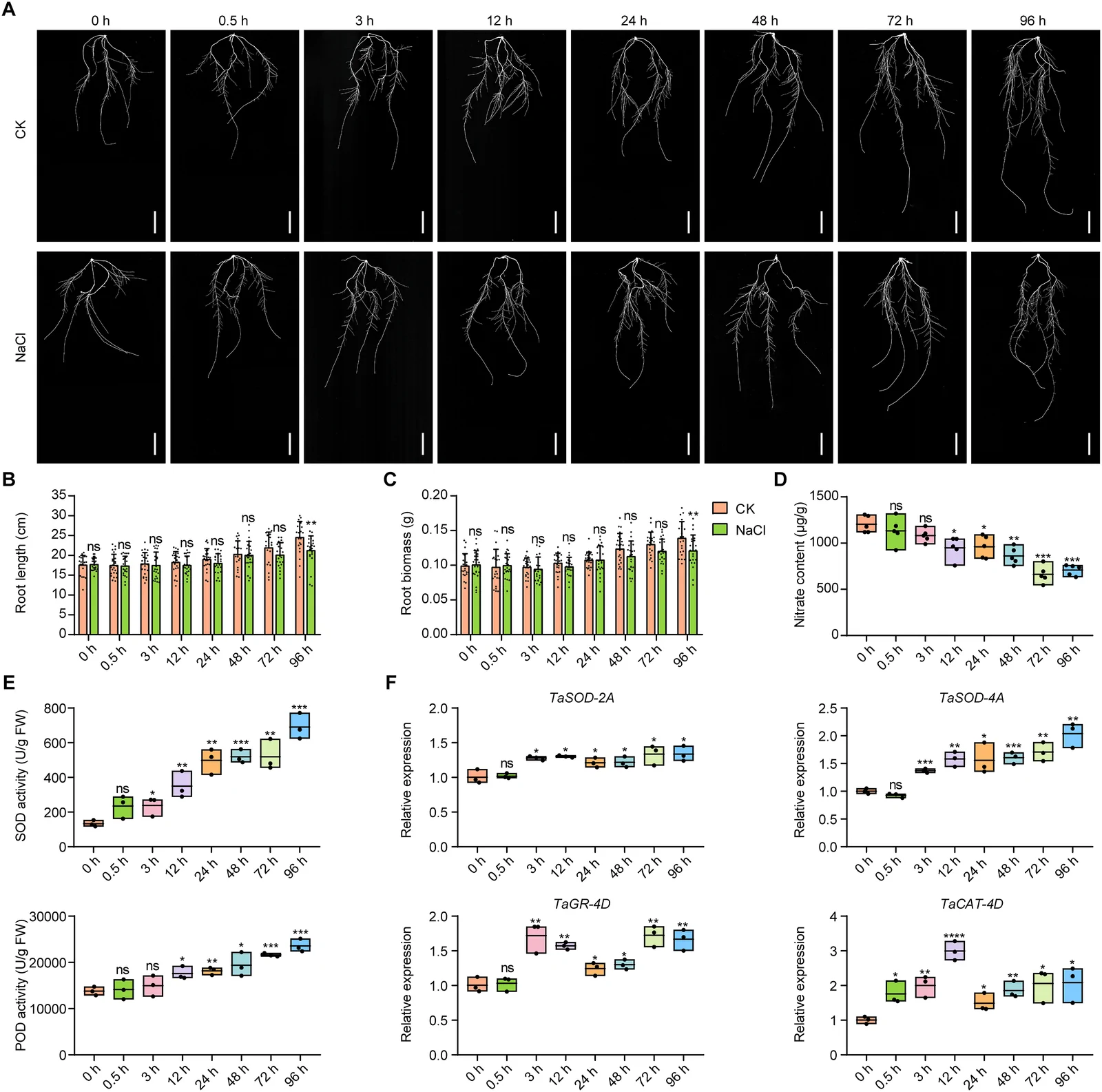
Effects of salt stress on root growth and nitrate content in wheat seedlings. **A** Morphological analyses of wheat roots after treatment with 0 or 100 mM NaCl for the indicated times. Plants were grown hydroponically and treated at the two-leaf-one-heart stage. Scale bar = 1 cm. **B–C** Statistical analyses of root length (**B**) and root biomass (**C**) for the seedlings shown in (**A**). Data are presented as means ± SD (*n* ≥ 10 biological replicates). **D–E** Physiological parameters of wheat seedlings under salt stress: nitrate content (**D**) and activities of the antioxidant enzymes superoxide dismutase (SOD) and peroxidase (POD) in roots (**E**). **F** Relative expression of genes encoding antioxidant enzymes in roots. Asterisks indicate statistically significant differences compared with the control. Data are presented as means ± SD (*n* ≥ 3 biological replicates). Asterisks indicate statistically significant differences determined by Student's *t*-test (∗∗∗*P* < 0.001, ∗∗*P* < 0.01, ∗*P* < 0.05; ns, not significant).

Given the essential role of nitrogen in plant growth and stress tolerance [13,24], we examined changes in nitrate content under salt stress to explore the underlying causes of this growth inhibition. Nitrate levels began to decline significantly after 12 h of treatment and continued to decrease with increasing treatment duration (Fig. 1D), demonstrating that salt stress impairs nitrate accumulation in wheat. Because nitrogen is a critical macronutrient for plant growth, this reduction in nitrate content likely contributes to the observed inhibition of root and shoot development.

In addition to nutritional imbalances, salt stress also triggers oxidative damage, necessitating the activation of antioxidant defenses. Accordingly, the activities of key antioxidant enzymes, including superoxide dismutase (SOD) and peroxidase (POD), increased markedly after 12 h of salt treatment (Fig. 1E), and the transcript levels of multiple genes encoding antioxidant enzymes, including *TaSOD-2A*, *TaSOD-4A*, *TaGR-4D*, and *TaCAT-4D*, were progressively upregulated (Fig. 1F). Taken together, these results demonstrate that salt stress suppresses nitrate accumulation, limiting nitrogen availability for plant growth while simultaneously activating the antioxidant defense system.

### 2.2 Salt stress induces rapid transcriptional reprogramming in wheat seedlings

To examine the molecular basis for the physiological responses observed above, we performed a transcriptome analysis of roots and shoots from wheat seedlings treated with 100 mM NaCl for 0, 0.5, 3, 12, 24, or 48 h. Principal component analysis (PCA) revealed clear separation of transcriptome profiles among treatment groups. In roots, PC1 and PC2 explained 23.59% and 16.02% of the total variance, respectively (Fig. 2A). Control samples were separated from salt-treated samples, indicating extensive transcriptional reprogramming in response to salinity. Samples from 12, 24, and 48 h clustered more closely, suggesting relatively minor transcriptional changes after prolonged treatment. In shoots, PC1 and PC2 accounted for 20.8% and 9.24% of the variance, respectively (Fig. S2A). Samples from 0 to 0.5 h clustered together, as did those from 24 to 48 h, indicating that major transcriptional shifts occurred between 3 and 24 h of salt treatment. We next examined overall patterns of gene expression using boxplots based on FPKM (fragments per kilobase of transcript per million mapped reads) values. Roots exhibited higher median gene-expression levels than shoots (Fig. 2B, Fig. S2B), revealing tissue-specific transcriptional activity consistent with their different responses to salt stress.

**Fig. 2:**
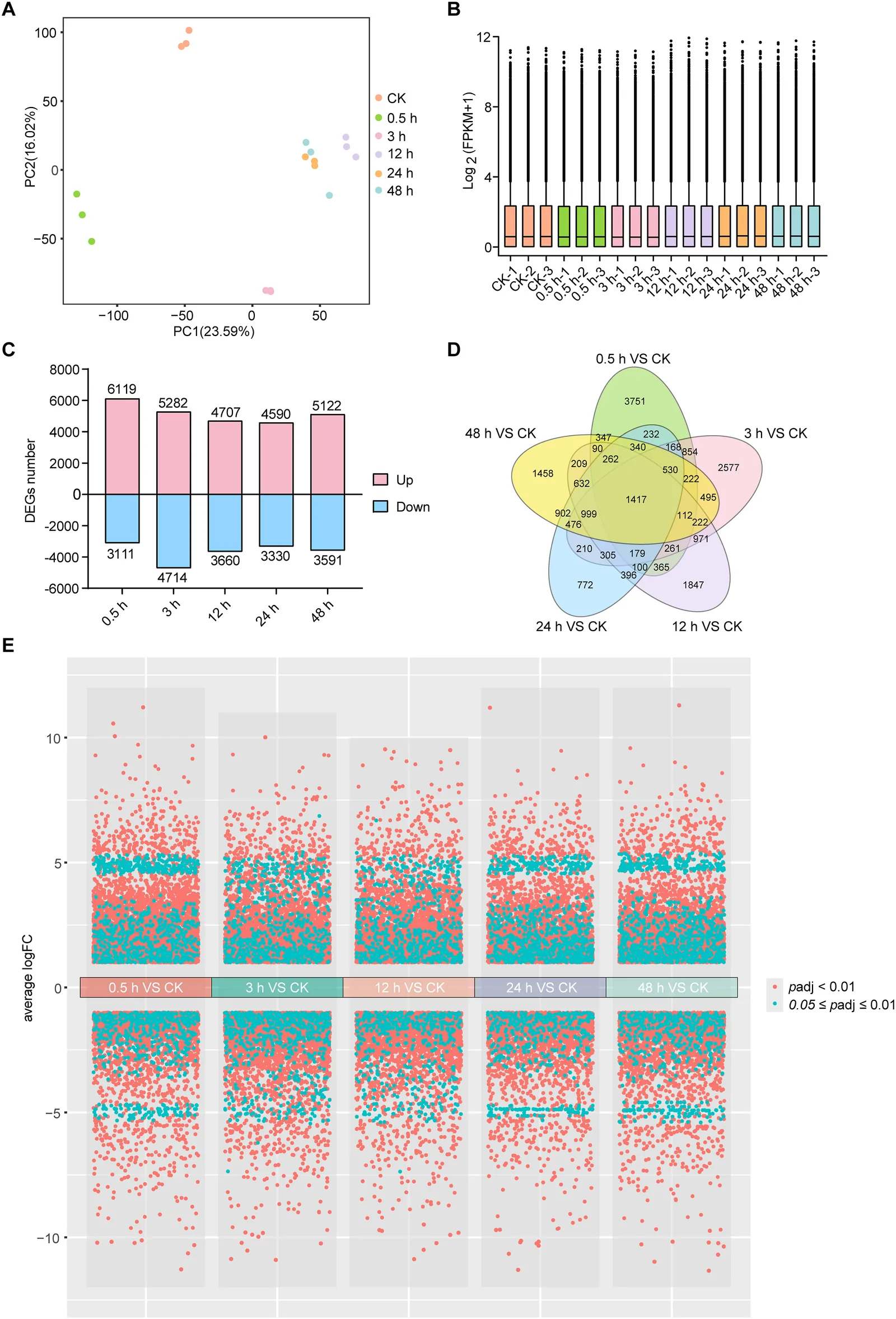
Transcriptional dynamics in wheat roots under salt stress. **A** Principal component analysis (PCA) of RNA-seq data from wheat roots treated with 100 mM NaCl for the indicated times. Each point represents a biological replicate, and colors indicate treatment durations. **B** Boxplots showing the distribution of FPKM values across all samples. The box represents the interquartile range, and the central line marks the median. The lower and upper hinges correspond to the first and third quartiles, respectively. **C** Numbers of upregulated and downregulated differentially expressed genes (DEGs) at each time point compared with the control (CK). DEGs were defined as genes with fold change ≥ 2 and adjusted *P*-value ≤ 0.05. **D** Venn diagram showing the overlap of DEGs identified in pairwise comparisons of different salt-treatment durations with the CK. **E** Volcano plots of DEGs identified in pairwise comparisons of different salt-treatment durations with the CK. Genes with adjusted *P* < 0.01 are highlighted in orange; genes with 0.01 ≤ adjusted *P* ≤ 0.05 are highlighted in blue.

Having examined global transcriptional activity, we next analyzed pairwise differences in gene expression between salt-treated and control samples at each time point. Genes with fold change ≥ 2 and adjusted *P* ≤ 0.05 were defined as differentially expressed genes (DEGs). We detected 9230, 9996, 8367, 7920, and 8713 DEGs in roots and 3527, 3076, 4926, 4204, and 3726 DEGs in shoots at 0.5, 3, 12, 24, and 48 h (Table S1–S2), respectively, relative to 0 h (Fig. 2C, Fig. S2C). A Venn diagram revealed shared and unique DEGs among the different treatment durations, including 1417 genes in roots and 259 genes in shoots that were differentially expressed in response to salt stress at all time points (Fig. 2D; Fig. S2D). Volcano plots were also constructed to visualize differential gene expression: points above the x axis represent upregulated genes, and those below represent downregulated genes (Fig. 2E; Fig. S2E).

Collectively, these results demonstrate that salt stress induces rapid and extensive transcriptional changes in both roots and shoots, with roots exhibiting an earlier and more pronounced response. The identified DEGs provide a foundation for further dissection of the molecular mechanisms that underlie salt-stress responses in wheat.

### 2.3 Transcriptional dynamics of salt-responsive genes reveal suppression of nitrate-related pathways

To further explore gene-expression dynamics in roots and shoots in response to salt treatment, we performed fuzzy c-means clustering of DEGs using the Mfuzz R package. We identified six expression clusters in both tissues, each of which exhibited unique temporal patterns following salt exposure (Fig. 3A and B; Fig. S3A–B). In roots, genes in clusters 1 and 4 showed rapid induction, with peak expression at 0.5 and 3 h, respectively. Genes in clusters 6 and 2 exhibited relatively stable expression during early treatment but were prominently upregulated at 12 and 24 h. Transcript levels of genes in cluster 3 continuously decreased throughout the time course, whereas those in cluster 5 showed a gradual increase (Fig. 3A). In shoots, genes in clusters 6, 1, 3, and 2 were upregulated in response to salt, reaching peak transcript levels at 0.5, 3, 12, and 48 h, respectively. Conversely, genes in clusters 4 and 5 were downregulated. Cluster 4 genes reached their lowest expression at 12 h before returning to baseline, whereas cluster 5 genes exhibited a sharp and sustained decline immediately following salt exposure (Fig. S3A).

**Fig. 3:**
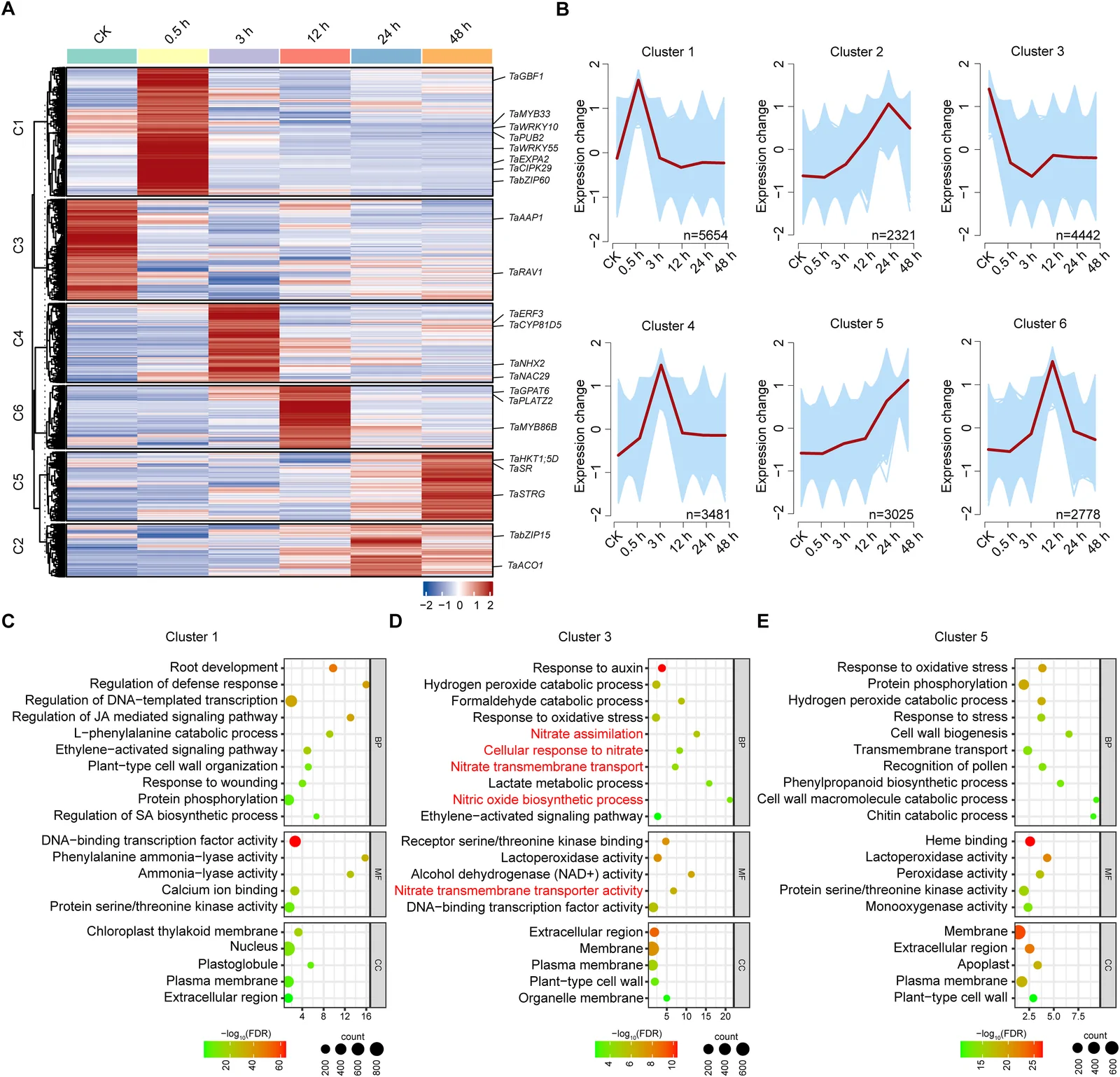
Clustering analysis and functional annotation of salt-responsive genes in wheat roots. **A** Heatmap showing the expression profiles of DEGs in roots exposed to different durations of salt treatment. Each row represents a gene, and each column represents a time point. Expression levels are *Z*-score normalized. **B** Fuzzy c-means clustering of DEGs using the Mfuzz R package. Each blue line depicts the expression pattern of one gene, and the cluster center for each group is shown in red. **C–E** Gene Ontology (GO) enrichment analysis of DEGs in cluster 1 (**C**), cluster 3 (**D**), and cluster 5 (**E**) as shown in (**B**). The top enriched biological process terms are displayed. In (**D**), terms related to nitrate assimilation and transport are highlighted in red.

We next performed Gene Ontology (GO) enrichment analysis on each cluster to gain insight into the biological processes associated with these gene-expression patterns. In roots, cluster 1 was enriched in genes related to root development, cell wall organization, and defense response, suggesting that these processes are rapidly regulated upon salt exposure (Fig. 3C). Other upregulated clusters (clusters 2, 4, 5, and 6) were predominantly associated with defense response and transmembrane transport (Fig. 3E; Fig. S4). Upregulated genes in shoots were enriched in comparable GO terms, suggesting that similar salt response mechanisms occur in roots and shoots (Fig. S3).

Among the downregulated gene clusters, cluster 4 in shoots was enriched in genes related to photosynthesis and light response, suggesting that these processes are suppressed by salt treatment, consistent with the observed changes in shoot morphology (Fig. S3F). Most notably, cluster 3 in roots and cluster 5 in shoots were significantly enriched in terms associated with nitrate assimilation, nitrate response, and nitrate transmembrane transport (Fig. 3D; Fig. S3G). This transcriptional suppression of nitrate-related genes is consistent with the reduced nitrate accumulation observed under salt stress (Fig. 1D) and likely contributes to impaired nitrogen metabolism and subsequent inhibition of seedling growth. Together, these results demonstrate that salt stress suppresses the expression of genes involved in nitrate acquisition and assimilation, providing a molecular basis for the reduced nitrate content and impaired plant development observed under saline conditions.

### 2.4 Key nitrate transporter and assimilation genes are downregulated under salt stress

In plants, nitrate is taken up and transported by NPF and NRT2 family transporters, then reduced to nitrite by NR and further to ammonium by nitrite reductase (NiR). Ammonium is subsequently assimilated into amino acids through the glutamine synthetase/glutamate synthase (GS/GOGAT) cycle [25]. Prompted by enrichment of nitrate-related terms among the downregulated gene clusters, we investigated how salt stress disrupts nitrogen metabolism in wheat seedlings by analyzing the expression of key nitrate transporter and assimilation genes using our RNA-seq datasets.

We first examined the expression patterns of *NPF* and *NRT2* family members, which encode the major nitrate transporters in plants. Heatmap visualization revealed distinct expression patterns of these genes under salt treatment (Fig. S5). *TaNRT1.1B* exhibited the highest basal expression and was markedly downregulated in response to salt stress. This suppression preceded or coincided with the decline in nitrate content, positioning *TaNRT1.1B* expression as an early molecular event in the salt-induced disruption of nitrogen metabolism and suggesting that it may play an important role in mediating nitrate uptake under saline conditions.

We next examined genes involved in nitrate assimilation. Transcript levels of *TaNIA* (encoding nitrate reductase), *TaNiR* (encoding nitrite reductase), and *TaGS2* (encoding glutamine synthetase) were significantly reduced under salt stress (Fig. S6A). These results indicate that salt treatment broadly suppresses the expression of genes involved in nitrate uptake and assimilation, impairing the efficient acquisition and metabolic utilization of nitrate in wheat seedlings.

To confirm the reliability of our RNA-seq data, we examined the expression of selected marker genes (*NRT1.1B*, *NIA1*, *NiR*, and *GS2*) by reverse transcription quantitative PCR (RT–qPCR). The expression patterns derived from RT–qPCR were highly consistent with those derived from the RNA-seq data across all time points (Fig. S6B), confirming the accuracy and reproducibility of our transcriptomic analyses. Taken together, these findings demonstrate that salt stress suppresses the transcription of genes central to nitrate transport and assimilation, providing a molecular explanation for the reduced nitrate accumulation and impaired nitrogen metabolism observed in salt-treated wheat seedlings (Fig. 1D).

### 2.5 Salt stress influences nitrate uptake by modulating TaNRT1.1B expression

Given the pronounced downregulation of *TaNRT1.1B* under salt stress (Fig. S6), we next investigated its role in mediating nitrate uptake and salt tolerance in wheat. Previous studies in *Arabidopsis* and rice have established NRT1.1 as a key regulator of nitrate signaling and transport. To characterize the function of *TaNRT1.1B* in wheat, we generated knockout mutants using CRISPR/Cas9 genome editing in the Fielder background.

Two single-guide RNAs (sgRNAs) were designed to target conserved regions in the exon of the three *TaNRT1.1B* homeologs (*TaNRT1.1B-A1*, -*B1*, and -*D1*) (Fig. 4A). After *Agrobacterium*-mediated transformation of immature Fielder embryos, we obtained two independent mutant lines, *tanrt1.1b-9* and *tanrt1.1b-18*. Sequencing of the target regions revealed distinct mutations in each homeolog (Fig. 4B–D). The *tanrt1.1b-9* line carried 1-bp insertions in *TaNRT1.1B-A1* and *TaNRT1.1B-B1* and a 249-bp deletion in *TaNRT1.1B-D1*. The *tanrt1.1b-18* line carried a 265-bp deletion in *TaNRT1.1B-A1*, a 1-bp insertion in *TaNRT1.1B-B1*, and a 249-bp deletion in *TaNRT1.1B-D1*. All mutations were predicted to cause a loss of gene function.

**Fig. 4:**
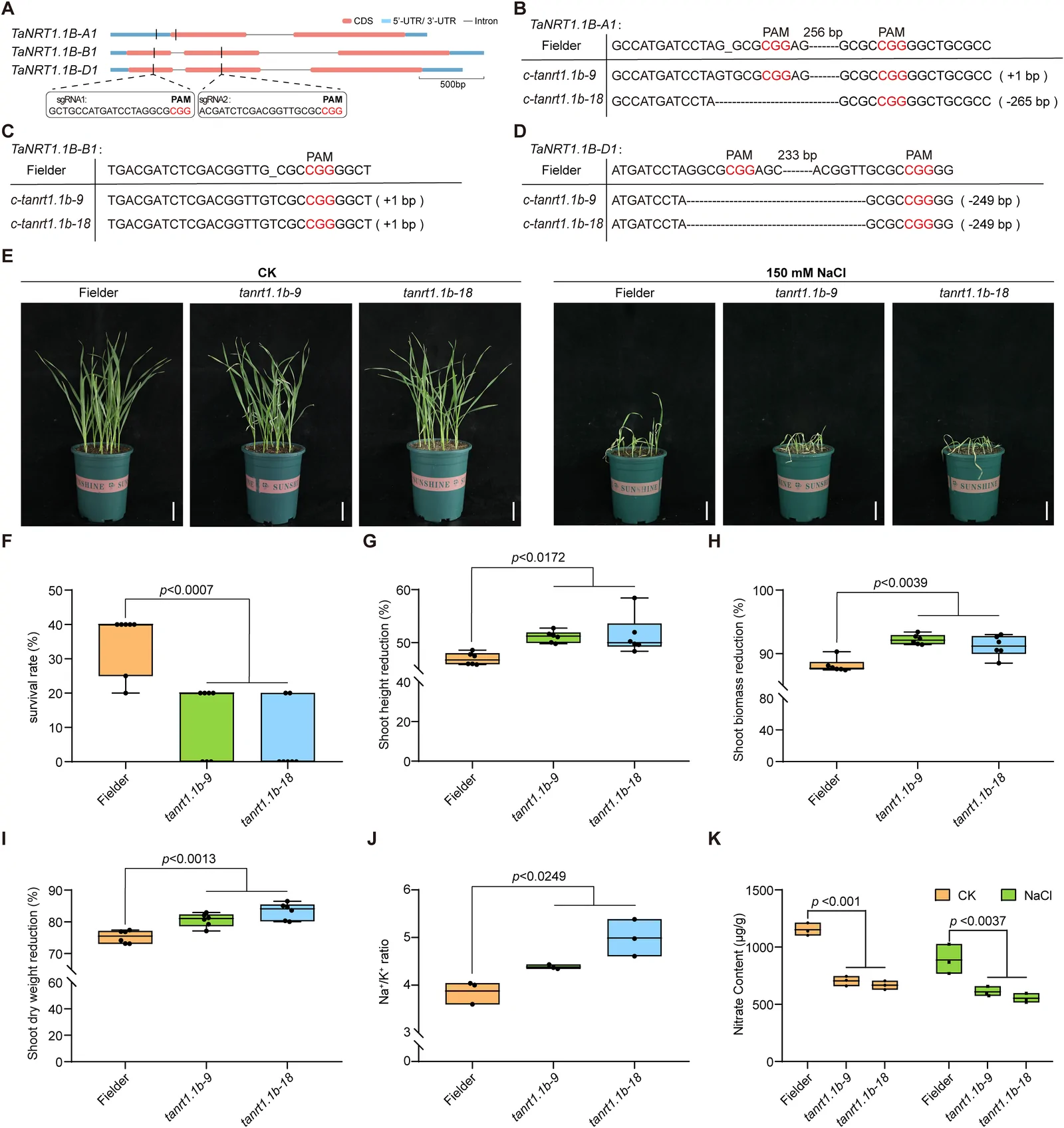
*TaNRT1.1B* positively regulates salt tolerance in wheat. **A** Diagram showing the positions of two single-guide RNAs (sgRNAs) targeting the *TaNRT1.1B* genomic region. Exons are represented by red boxes, and introns are represented by lines. The 5′ UTR and 3′ UTR are indicated by blue boxes. **B–D** Generation of *tanrt1.1b* mutants in the Fielder background using CRISPR/Cas9 genome editing. Sequence alignments show mutations in the target sites of three *TaNRT1.1B* homeologs in two independent mutant lines (*tanrt1.1b-9* and *tanrt1.1b-18*): *TaNRT1.1B-A1* (**B**), *TaNRT1.1B-B1* (**C**), and *TaNRT1.1B-D1* (**D**). **E** Morphological analysis of wild-type Fielder and *tanrt1.1b* mutants under control conditions (left panels) and after treatment with 150 mM NaCl for 14 days (right panels). Treatments were initiated at the one-leaf-one-heart stage. Scale bar = 5 cm. **F–J** Statistical analysis of survival rate (**F**), shoot height reduction (**G**), shoot biomass reduction (**H**), shoot dry weight reduction (**I**), and the Na^+^/K^+^ ratio (**J**) of the seedlings shown in (**E**). **K** Nitrate content in wild-type Fielder and *tanrt1.1b* mutants under control and salt-treated conditions. Data are presented as means ± SD (*n* ≥ 3 biological replicates). Statistically significant differences were determined by Student's *t*-test.

We next examined the response of *tanrt1.1b* mutants to salt stress. Upon salt treatment, germination and survival rates were significantly lower in the *tanrt1.1b* mutants than in the wild-type (Fig. 4E, F and Fig. S7). Salt stress caused greater reductions in shoot height, shoot biomass, and shoot dry weight in the mutants than in wild-type plants (Fig. 4G–I), indicating that disruption of *TaNRT1.1B* compromises plant growth under saline conditions.

To explore the physiological basis of this heightened sensitivity, we measured Na^+^ and K^+^ contents in wild-type plants and *tanrt1.1b* mutants. The Na^+^/K^+^ ratio was significantly higher in the mutants under salt stress (Fig. 4J), consistent with their more severe phenotypic changes, and nitrate content was significantly lower in *tanrt1.1b* mutants than in wild-type plants under both control and salt-treated conditions (Fig. 4K). Collectively, these results demonstrate that *TaNRT1.1B* positively regulates salt tolerance in wheat by sustaining nitrate uptake and contributing to ionic homeostasis under saline conditions.

### 2.6 Mutation of TaNRT1.1B affects ROS accumulation in wheat

Long-term exposure to high-salt environments induces excessive production of ROS, which cause oxidative damage to lipids, proteins, and nucleic acids [26,27]. To examine whether *TaNRT1.1B* mediates oxidative stress responses under salt stress, we investigated ROS accumulation and antioxidant enzyme activities in wild-type and *tanrt1.1b* mutant wheat lines. Histochemical staining with nitroblue tetrazolium (NBT) and 3,3′-diaminobenzidine (DAB) revealed substantially stronger signals of O_2_^−^ and H_2_O_2_ in the *tanrt1.1b* mutants under salt stress (Fig. 5A and B). Accordingly, levels of both H_2_O_2_ and O_2_^−^ were significantly higher in the *tanrt1.1b* mutants than in the WT, whereas SOD and POD activities were much lower (Fig. 5C–F).

**Fig. 5:**
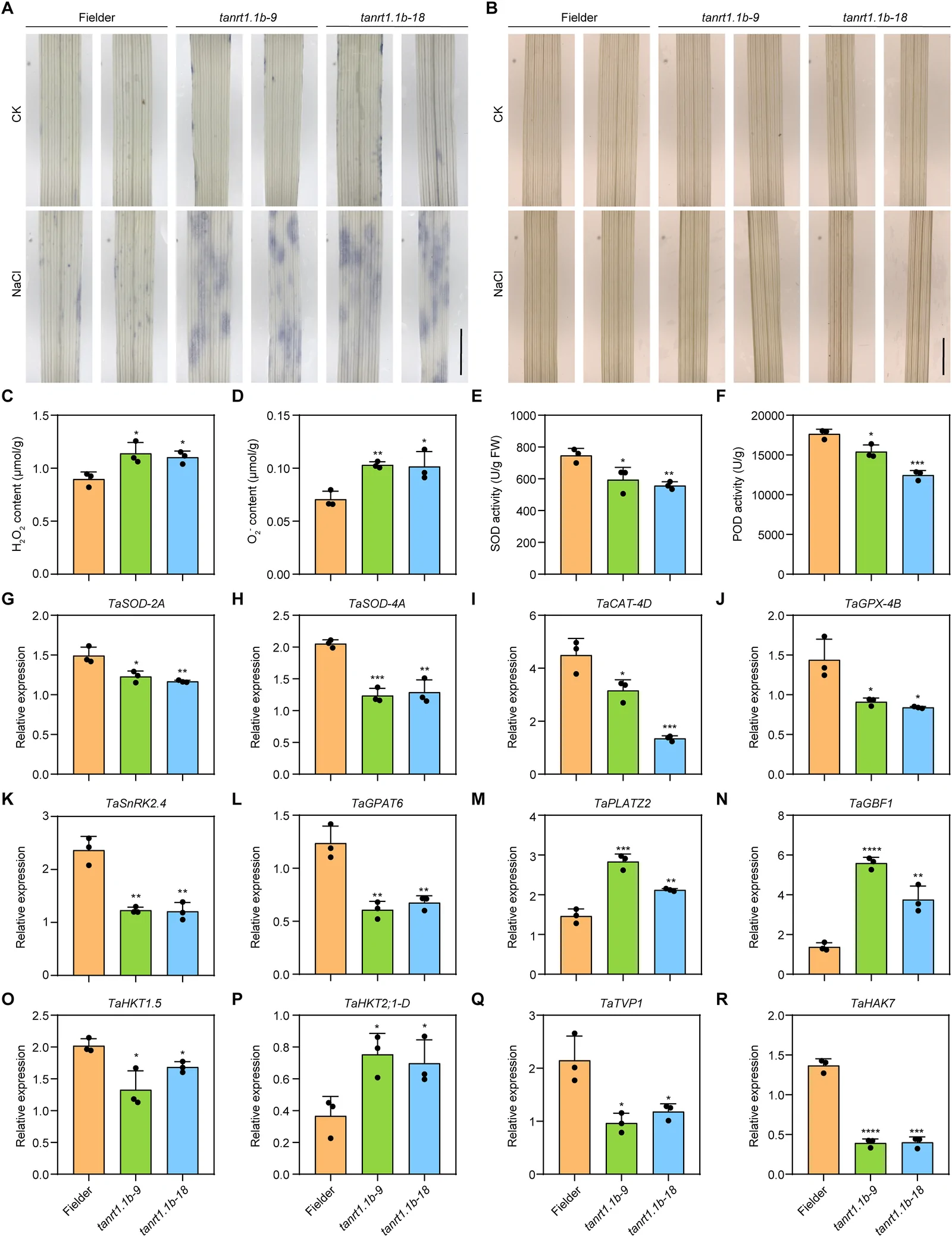
Changes in antioxidant enzyme activities and related gene expression in wild-type Fielder and *tanrt1.1b* mutants. **A–B** NBT (**A**) and DAB (**B**) staining of wild-type plants and *tanrt1.1b* mutants under normal and salt-stress conditions. Plants were grown in soil to the one-leaf-one-heart stage, then exposed to 0 or 150 mM NaCl for 7 days. Scale bar = 200 μm. **C–F** Physiological characterization of hydroponically grown wheat seedlings under salt stress: quantitative measurements of root H_2_O_2_ content (**C**), O_2_^−^ content (**D**), SOD activity (**E**), and POD activity (**F**). Plants were grown hydroponically and treated at the two-leaf-one-heart stage. **G–J** Relative expression levels of genes encoding antioxidant enzymes in roots. **K–N** Relative expression of genes involved in salt-tolerance regulation. **O–R** Relative expression of genes related to Na^+^ and K^+^ transport. Data are presented as the means ± SD (*n* = 3 biological replicates). Asterisks indicate statistically significant differences determined by Student's *t*-test (∗∗∗∗*P* < 0.0001, ∗∗∗*P* < 0.001, ∗∗*P* < 0.01, ∗*P* < 0.05).

We next assessed the expression of genes related to ROS detoxification. The *tanrt1.1b* mutants exhibited significant downregulation of *TaSOD-4A*, *TaCAT-4D*, *TaSOD-2A*, *TaGPX-4B*, *TaGR-4D*, and *TaAPX-2B*, which may have caused excessive ROS accumulation and ultimately impaired salt tolerance (Fig. 5G–J and Fig. S8A and B). These results are in agreement with the enzyme activity assays. However, given that *TaNRT1.1B* functions in nitrate acquisition rather than directly in ROS detoxification or transcriptional regulation, these transcriptional changes likely reflect secondary consequences of altered nitrogen status and associated metabolic or hormonal reprogramming.

To gain further insight into why ROS scavenging–related genes were downregulated in the *tanrt1.1b* mutants, we analyzed the expression of reported salt-tolerance regulatory genes in wheat. Positive regulators of salt tolerance such as *TaSnRK2.4*, *TaGPAT6*, *TaSK5*, *TaFER5D-1*, and *TaPRX-2A* were significantly downregulated in the *tanrt1.1b* mutants, whereas negative regulators such as *TaGBF1* and *TaPLATZ2* were markedly upregulated (Fig. 5K–N and Fig. S8C–J).

To investigate the molecular basis for the elevated Na^+^/K^+^ ratio in the *tanrt1.1b* mutants, we examined the expression of genes related to Na^+^ and K^+^ transport in wheat. Genes associated with Na^+^ efflux (*TaHKT1;5*), Na^+^ extrusion (*TaSOS1*), and vacuolar sequestration (*TaTVP1*) were down-regulated in the *tanrt1.1b* mutants, as were genes related to K^+^ uptake (e.g. *TaHAK7* and *TaHAK9*); collectively, these changes likely contributed to the increased Na^+^/K^+^ ratio (Fig. 5O–R and Fig. S8K–P). These concerted changes in the transcription of genes associated with salt-tolerance regulation, ROS scavenging, and Na^+^/K^+^ transport likely underlie the reduced ROS detoxification capacity and disrupted ionic homeostasis observed in the *tanrt1.1b* mutants, ultimately accounting for their salt-sensitive phenotype.

### 2.7 TaNRT1.1B contributes to ABA sensitivity in wheat

Rice OsNRT1.1B acts as an ABA receptor at low ABA concentrations and is essential for the modulation of ABA signaling [24]. To explore whether *TaNRT1.1B* plays a similar conserved role in wheat, we sowed surface-sterilized seeds of wild-type and *tanrt1.1b* mutant lines in germination pouches supplemented with 0 or 200 nM ABA and grew them for 4 days for phenotypic analyses. In the absence of exogenous ABA, there were no significant differences in seedling or root growth between the *tanrt1.1b* mutants and wild-type plants. ABA treatment significantly reduced root length in wild-type plants, but this inhibitory effect was largely abolished in the *tanrt1.1b* mutants (Fig. 6A–C), indicating that the mutants were insensitive to ABA.

**Fig. 6:**
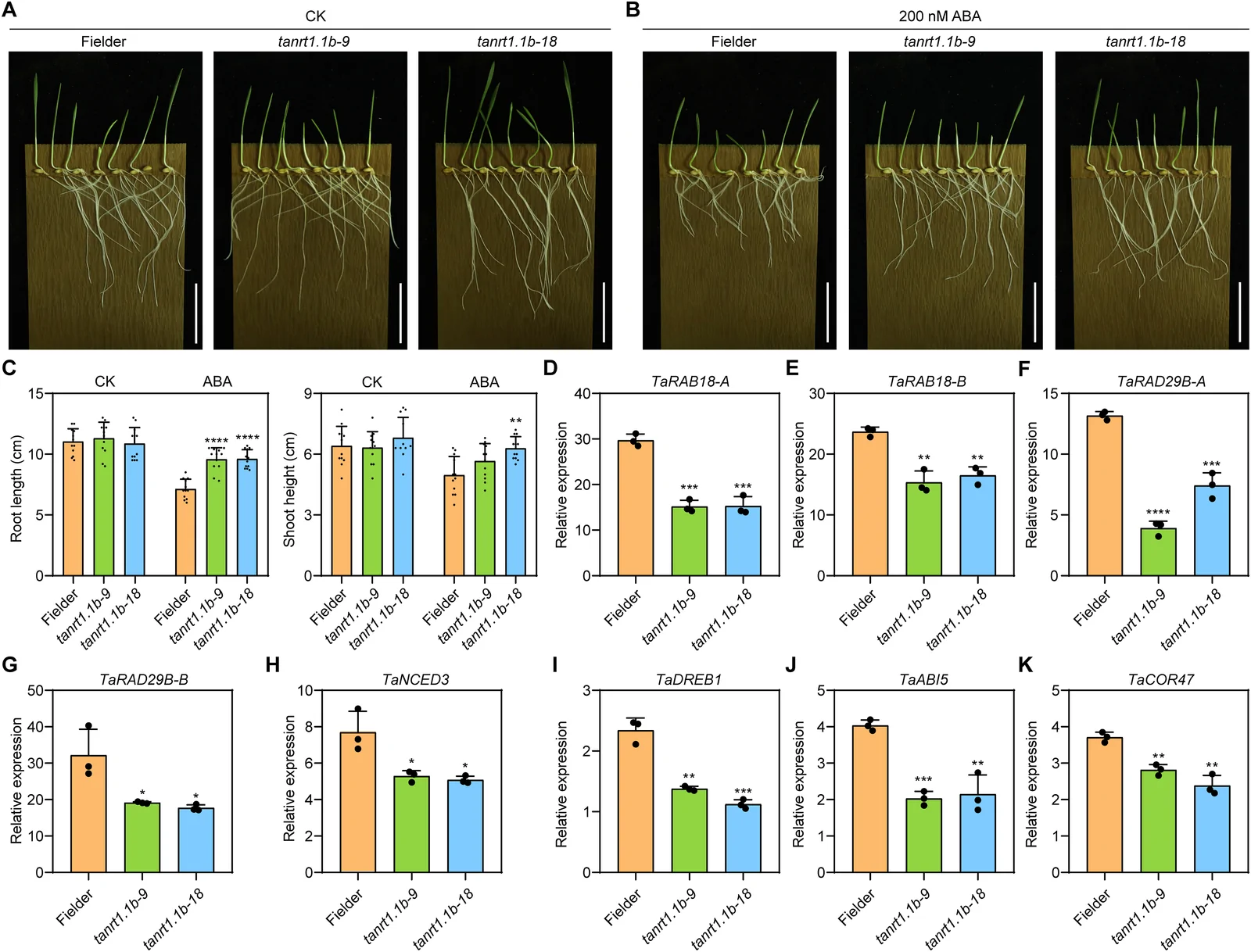
*TaNRT1.1B*-mediated regulation of ABA responses in wheat. **A–B** Morphological analysis of wheat seedlings treated with 0 (**A**) or 200 nM ABA (**B**) for 4 days in germination pouches. Scale bar = 5 cm. **C** Root length and shoot height of the seedlings shown in (**A**) and (**B**). Data are presented as means ± SD (*n* = 10 biological replicates). **D–K** Relative expression of ABA-responsive genes in roots. Seedlings at the two-leaf-one-heart stage were treated with 0 or 200 nM ABA for 2 h. Data are presented as means ± SD (*n* = 3 biological replicates). Asterisks indicate statistically significant differences determined by Student's *t*-test or two-way ANOVA (∗∗∗∗*P* < 0.0001, ∗∗∗*P* < 0.001, ∗∗*P* < 0.01, ∗*P* < 0.05).

To further investigate the role of *TaNRT1.1B* in ABA responses, we examined the expression of ABA-responsive genes by RT–qPCR [28,29]. *TaRAB18-A*, *TaRAB18-B*, *TaRAD29B-A*, *TaRAD29B-B*, *TaNCED3*, *TaDREB1*, *TaABI5*, and *TaCOR47* were significantly downregulated in the *tanrt1.1b* mutants compared with the wild-type (Fig. 6D–K), consistent with their ABA-insensitive growth phenotype. These results suggest that *TaNRT1.1B* may participate in mediating wheat responses to low ABA concentrations, a role analogous to that proposed for *OsNRT1.1B* in rice.

### 2.8 Loss of TaNRT1.1B function significantly reduced wheat yield traits under salt stress

Having established that *TaNRT1.1B* positively regulates vegetative growth under salt stress (Fig. 4), we next asked whether it also influences reproductive development and yield traits. To this end, we grew wild-type and *tanrt1.1b* plants with or without 75 mM NaCl starting at the one-leaf-one-heart stage, then performed phenotypic analyses at maturity. Under control conditions, the *tanrt1.1b* mutants initiated heading earlier than wild-type plants. Both genotypes reached heading under salt stress, but the mutants completed heading earlier than the wild-type (Fig. 7A). These observations suggest that salt stress accelerates wheat heading and that this effect is exacerbated in the absence of functional *TaNRT1.1B*.

**Fig. 7:**
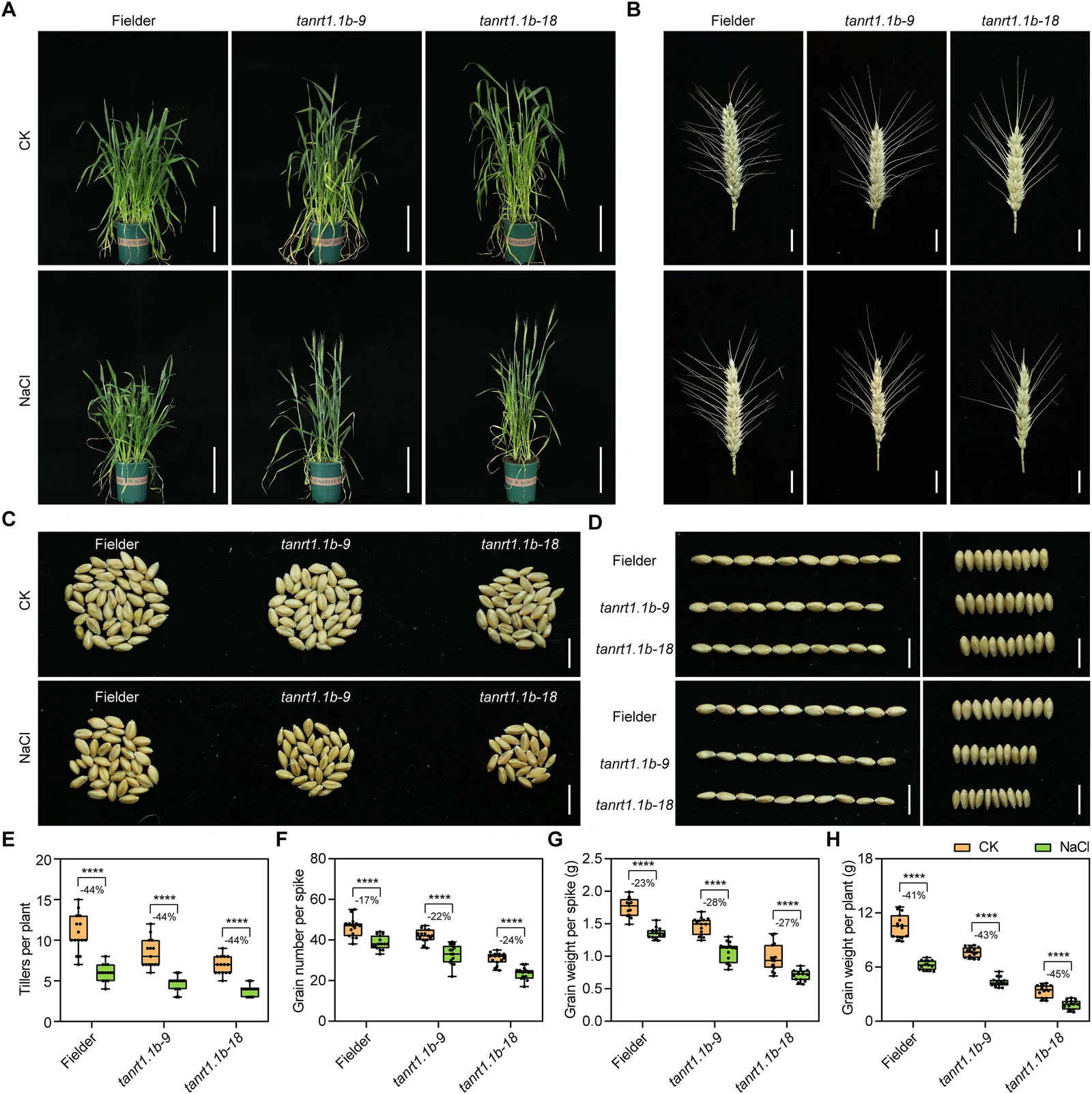
*TaNRT1.1B* positively regulates wheat yield traits under salt stress. **A** Phenotypic analysis of wild-type Fielder and *tanrt1.1b* mutants under control conditions and after 45 days of salt treatment (75 mM NaCl beginning at the one-leaf-one-heart stage). Scale bar = 20 cm. **B** Morphological analysis of spikes from Fielder and *tanrt1.1b* mutants grown under control and salt-treated conditions and harvested at maturity. Wheat seedlings were treated with 75 mM NaCl beginning at the one-leaf-one-heart stage. Scale bar = 2 cm. **C–D** Phenotypic characterization of grain number per spike (**C**) and grain morphology (**D**) in wild-type Fielder and *tanrt1.1b* mutants. (**D**) shows representative grains (10 grains per line). Scale bars = 1 cm. **E–H** Statistical analysis of tiller number per plant (**E**), grain number per spike (**F**), grain weight per spike (**G**), and grain weight per plant (**H**) of the plants shown in (**A–B**). Data are presented as means ± SD (*n* = 15). Asterisks indicate statistically significant differences determined by two-way ANOVA (∗∗∗∗*P* < 0.0001, ∗∗∗*P* < 0.001, ∗∗*P* < 0.01).

We next examined yield-related traits in mature plants. The spike length of *tanrt1.1b* mutants was significantly reduced compared with that of wild-type plants under both control and salt-stressed conditions (Fig. 7B). The grain number per spike decreased by 17% in the WT and by 22% and 24%, in the mutants under salt stress (Fig. 7C–F). Under salt treatment, both grain length and grain width were significantly lower in *tanrt1.1b* mutants than in wild-type plants (Fig. 7D). Consistent with these observations, grain weight per spike and grain weight per plant were significantly lower in the mutants under both control and saline conditions (Fig. 7G and H). The reductions in yield-related traits observed in *tanrt1.1b* mutants under salt stress are consistent with the impaired vegetative growth and reduced nitrate accumulation observed at the seedling stage, suggesting that the function of *TaNRT1.1B* is essential for maintaining productivity under saline conditions throughout development.

### 2.9 Salt-tolerant wheat accessions maintain higher TaNRT1.1B expression and nitrate levels

The observed contribution of *TaNRT1.1B* to nitrate uptake and salt tolerance (Fig. 4, Fig. 5, Fig. 6, Fig. 7) prompted us to examine whether natural variation in salt tolerance among wheat accessions was associated with differences in nitrate accumulation and *TaNRT1.1B* expression. To this end, we screened a panel of wheat varieties under control and salt-stress conditions (100 mM NaCl for 21 days) and selected representative salt-tolerant and salt-sensitive lines for detailed phenotypic and molecular analyses. Morphological assessment revealed striking differences between the two groups under salt treatment (Table S2). The salt-tolerant accessions maintained relatively healthy shoot growth with minimal wilting and chlorosis, whereas the salt-sensitive accessions exhibited severe growth inhibition, pronounced leaf wilting, and reduced overall vigor (Fig. 8A and B). Quantitative analysis confirmed that salt-induced reductions in shoot biomass were significantly greater in the salt-sensitive lines than in the salt-tolerant lines (Fig. 8C).

**Fig. 8:**
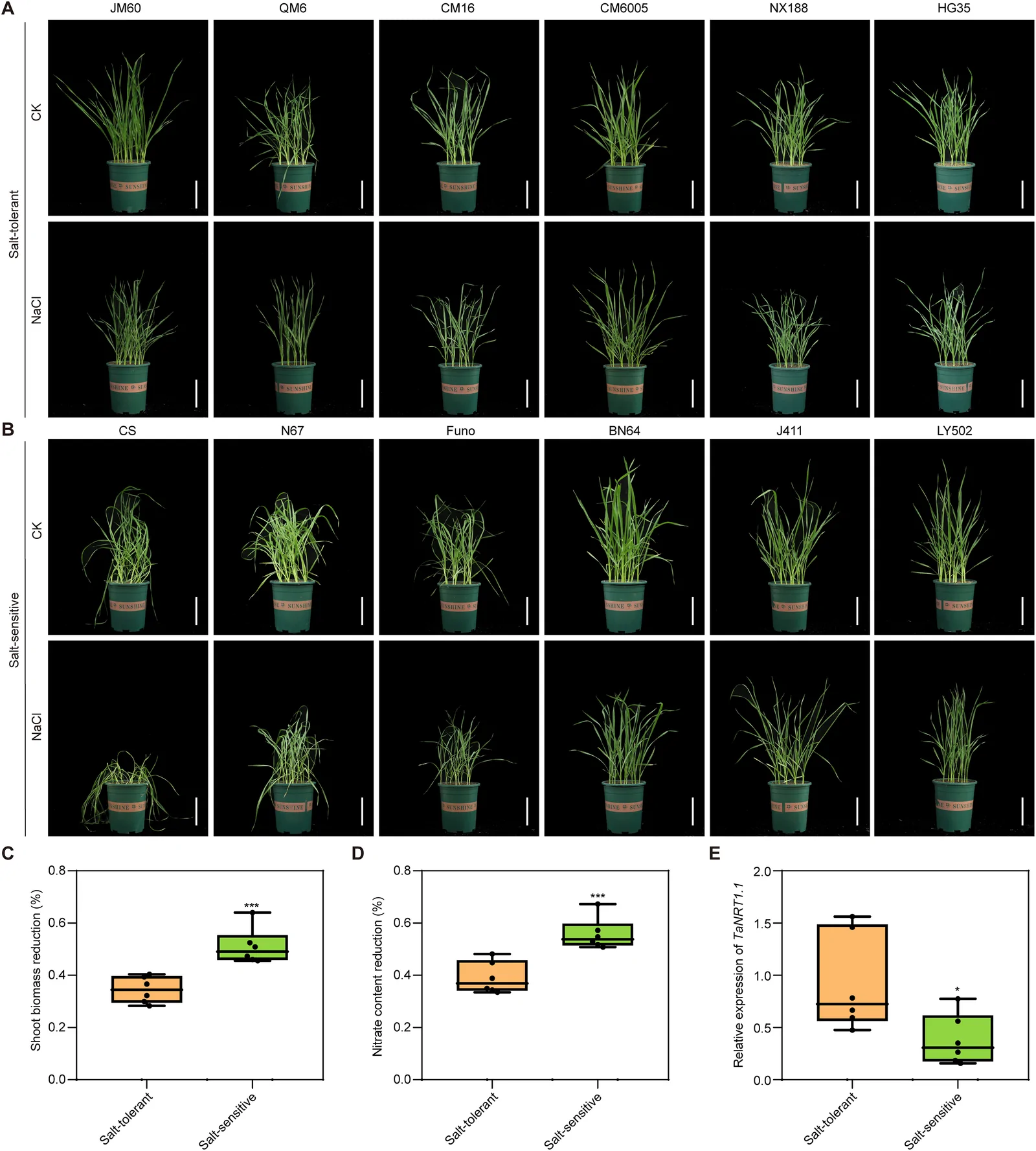
Association between salt tolerance and nitrate accumulation in wheat. **A–B** Morphological analysis of representative salt-tolerant (**A**) and salt-sensitive (**B**) wheat accessions after treatment with 0 or 100 mM NaCl for 21 days. Treatments were initiated at the one-leaf-one-heart stage. Scale bar = 10 cm. **C–D** Statistical analysis of shoot biomass reduction (**C**) and nitrate content reduction (**D**) under salt stress in the wheat accessions shown in (**A–B**). **E** Relative expression level of *TaNRT1.1B* in roots of the salt-tolerant and salt-sensitive wheat accessions shown in (**A–B**). Data are presented as means ± SD (*n* ≥ 3 biological replicates). Asterisks indicate statistically significant differences determined by Student's *t*-test (∗∗∗*P* < 0.001, ∗*P* < 0.05).

To explore whether these different responses were associated with nitrate metabolism, we measured nitrate content in the shoots of both groups under control and salt-stressed conditions. Salt-sensitive lines showed a significantly greater reduction in nitrate content than salt-tolerant lines under salt stress (Fig. 8D), suggesting that enhanced nitrate retention or uptake capacity may contribute to their greater salt tolerance. Given the established role of TaNRT1.1B in nitrate uptake, we examined its transcript levels in roots of these accessions under salt stress. RT–qPCR analysis revealed that *TaNRT1.1B* expression was significantly higher in the salt-tolerant accessions than in the salt-sensitive accessions after salt treatment (Fig. 8E). This positive association between *TaNRT1.1B* transcript abundance and salt tolerance provides further support for the notion that *TaNRT1.1B*-mediated nitrate uptake plays a protective role under saline conditions. Taken together, these results demonstrate that *TaNRT1.1B* expression promotes salt tolerance in wheat, likely by maintaining nitrate acquisition and contributing to ionic homeostasis under salt stress.

## 3 Discussion

Soil salinity is a major environmental constraint that severely inhibits plant growth and reduces crop yields worldwide [5,30]. High salinity leads to hyperosmotic stress and ion toxicity, thereby suppressing water and nutrient uptake [5,24]. Although the physiological consequences of salt stress are well documented, the molecular mechanisms by which salt stress affects nitrogen metabolism and plant development remain poorly understood in wheat. In this study, we demonstrated that salt stress inhibits wheat seedling growth, reduces nitrate accumulation, and suppresses the expression of genes involved in nitrate transport and assimilation (Fig. 1, Fig. 3 and Fig. S6). Through combined transcriptomic, genetic, and molecular analyses, we identified *TaNRT1.1B* as a key regulator that links nitrate acquisition and salt-tolerance pathways, coordinating nutrient status and stress responses to modulate wheat growth and yield.

### 3.1 Salt stress disrupts nitrogen metabolism at the physiological and transcriptional levels

Soil salinity and nutrient bioavailability are critical environmental factors that jointly influence plant growth and development. Nitrogen fertilization has been shown to enhance plant salt tolerance on saline soils [31,32]. However, the molecular basis for this beneficial effect has not been fully clarified in wheat. Here, we observed that nitrate content declines significantly under prolonged salt treatment (Fig. 1D). Transcriptome profiling revealed that genes associated with nitrate transport (*NPF* and *NRT2* families) and assimilation (*NIA*, *NiR*, and *GS*) are progressively downregulated under salt stress (Fig. 3, Fig. S5 and S6), providing a molecular basis for the observed reduction in nitrate accumulation. Enrichment of nitrate-related terms among downregulated genes in both roots and shoots indicates that the suppression of nitrogen metabolism in response to salinity occurs across tissues. This nitrogen deficit likely contributes to the observed growth inhibition, as nitrogen is essential for the biosynthesis of amino acids, proteins, and other nitrogen-containing compounds [14,25].

### 3.2 TaNRT1.1B is associated with altered ROS accumulation under salt stress

The NRT1.1 family has been studied extensively in *Arabidopsis* and rice. In these species, NRT1.1 functions as both a nitrate transporter and a sensor, modulating nitrate signaling, plant growth, and stress responses [20,24,33]. In wheat, we observed that *TaNRT1.1B* is strongly downregulated under salt stress (Fig. S6) and that its disruption reduces nitrate content, increases the Na^+^/K^+^ ratio, and enhances salt sensitivity (Fig. 4). These phenotypes are consistent with the notion that *TaNRT1.1B*-mediated nitrate uptake contributes to ionic homeostasis under saline conditions.

Salt stress induces the production of various ROS in plants, including the superoxide anion (O_2__⁠_^−^), hydrogen peroxide (H_2_O_2_), singlet oxygen (^1^O_2_), and the hydroxyl radical (·OH). In addition to causing oxidative damage, such ROS can also function as signaling effectors in plant stress-adaptation mechanisms [[34], [35], [36]]. Numerous protein kinases, transcription factors, and key enzymes play pivotal roles in the regulation of ROS accumulation and scavenging [[37], [38], [39], [40]]. Our results showed that loss of *TaNRT1.1B* function led to downregulation of multiple genes encoding ROS scavenging enzymes and salt-tolerance regulators, and this was accompanied by reduced antioxidant enzyme activities (Fig. 5). Given that *TaNRT1.1B* primarily functions in nitrate acquisition, these transcriptional changes likely reflect secondary consequences of altered nitrogen status rather than direct regulation by *TaNRT1.1B*.

### 3.3 Like its rice ortholog, wheat TaNRT1.1B is involved in ABA responses

Rice OsNRT1.1B has been shown to function as an ABA receptor, playing an essential role in modulating ABA signaling [24]. Our findings reveal that this function is conserved in wheat, as *tanrt1.1b* mutants exhibited pronounced ABA insensitivity in root growth assays and failed to fully induce ABA-responsive genes such as *TaRAB18*, *TaRAD29B*, *TaABI5*, and *TaCOR47* upon ABA treatment (Fig. 6). This discovery not only establishes *TaNRT1.1B* as a dual-function protein involved in both nitrate transport and ABA perception but also suggests that the ABA signaling module may serve as an additional point of crosstalk through which nitrogen status modulates stress adaptation. Given that ABA is a central regulator of salt and drought responses, the impaired ABA sensitivity of the *tanrt1.1b* mutants likely exacerbates their salt-sensitive phenotype, linking the nitrate transport function of TaNRT1.1B to broader hormonal stress networks.

### 3.4 TaNRT1.1B links nitrate uptake with developmental and yield responses under salt stress

The improvement of crop NUE is critical for tackling salinity-related challenges and increasing sustainable crop production. Soil nitrogen deficiency limits primary metabolic activities in plants, reducing rates of both transpiration and photosynthesis and thus compromising plant growth. Notably, flowering is accelerated under low-nitrogen conditions. Nitrogen deficiency causes the most significant yield losses when it occurs at the tillering stage [[41], [42], [43]]. Consistent with these observations, our results demonstrated that salt stress induces early heading in wheat and that this effect is exacerbated by *TaNRT1.1B* mutation. Moreover, this mutation significantly reduced spike length, grain number per spike, and grain weight per plant in wheat (Fig. 7). These findings suggest that salt stress may suppress the expression of *TaNRT1.1B*, thereby compromising NUE and ultimately reducing wheat yield.

### 3.5 Implications for breeding of salt-tolerant wheat varieties

The identification of *TaNRT1.1B* as a key determinant of salt tolerance and nitrogen uptake suggests that it may be a valuable genetic target for wheat improvement. The positive association between *TaNRT1.1B* expression and salt tolerance in diverse wheat accessions (Fig. 8) suggests that natural or engineered variation in this gene could be exploited to enhance stress resilience. However, several questions remain to be addressed. First, our finding that *tanrt1.1b* mutants are insensitive to ABA suggests that *TaNRT1.1B* functions in both nitrate transport and ABA signaling. However, the underlying mechanism by which *TaNRT1.1B* integrates these two functions under saline conditions requires further investigation. Second, the downstream targets and interaction partners of *TaNRT1.1B* remain to be identified, as do natural variations and superior haplotypes of *TaNRT1.1B* in diverse germplasm. Follow-up studies will expand the screening of natural variations to further evaluate their potential for molecular breeding. Lastly, the effect of *TaNRT1.1B* manipulation on wheat performance under field conditions and its effect on yield under combined stresses (e.g., salinity and nitrogen deficiency) remain to be evaluated.

In summary, this study identifies *TaNRT1.1B* as a key determinant of both salt tolerance and nitrogen acquisition. By coupling nitrate uptake with salt tolerance, *TaNRT1.1B* modulates wheat growth, stress tolerance, and yield-related traits (Fig. 9). These findings advance our understanding of the molecular mechanisms that link nutrient status and stress responses in crops and provide a potential genetic target for breeding of wheat varieties with enhanced resilience to abiotic stress, improved NUE, and stable grain yields.

**Fig. 9:**
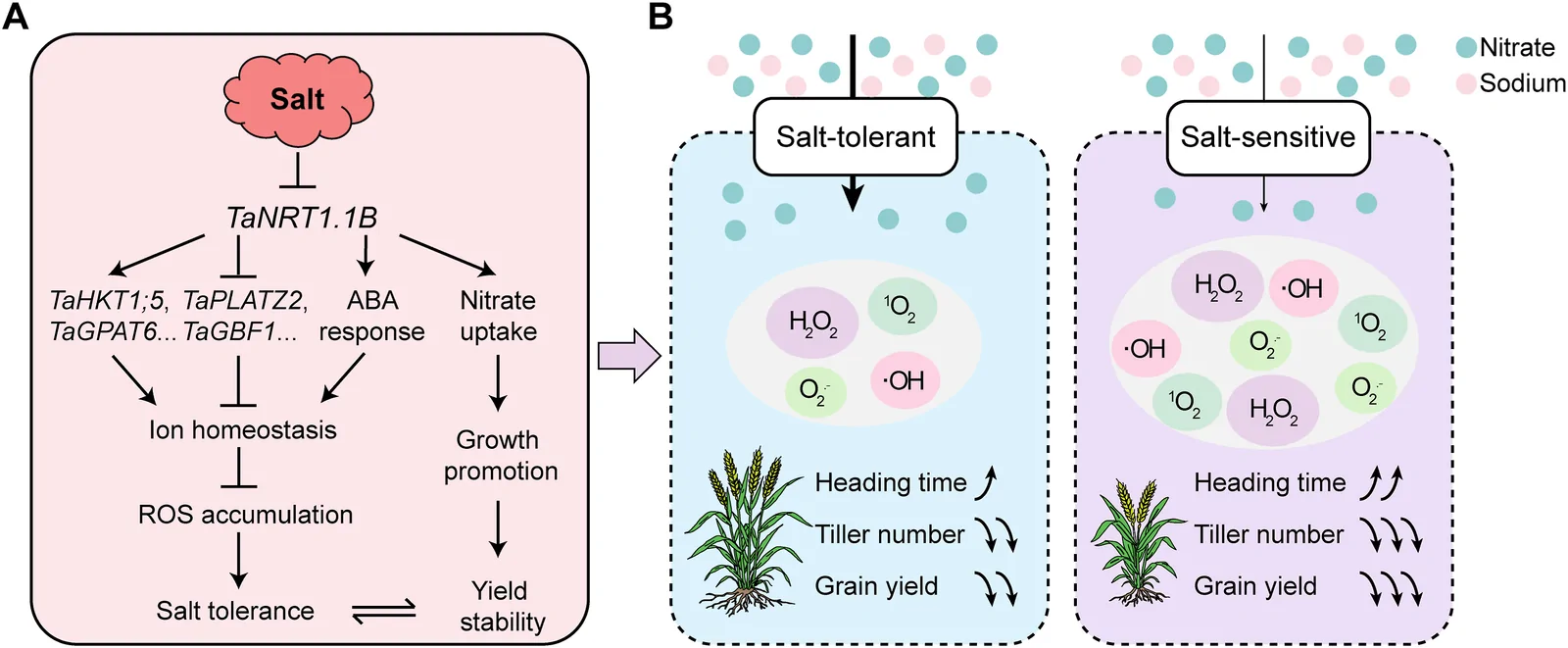
A working model explaining how *TaNRT1.1B* links nitrate uptake to salt tolerance in wheat. **A** Under salt stress, *TaNRT1.1B* expression is suppressed, and this is accompanied by coordinated changes in the expression of salt-stress regulatory genes, including both positive (e.g., *TaHKT1;5* and *TaGPAT6*) and negative regulators (e.g., *TaPLATZ2* and *TaGBF1*). Disruption of *TaNRT1.1B* also renders wheat insensitive to ABA. These transcriptional changes promote ROS accumulation and compromise salt tolerance. At the same time, reduced *TaNRT1.1B* expression impairs nitrate acquisition from the environment, restricting plant growth and ultimately reducing grain yield. **B** Under salt stress, salt-tolerant accessions exhibit higher *TaNRT1.1B* expression than salt-sensitive accessions and thus maintain higher nitrate levels. This higher nitrate status likely helps to maintain ROS detoxification capacity and alleviates developmental impairments, contributing to higher values of yield components such as tiller number and heading time and ultimately to greater yield stability relative to salt-sensitive varieties. These results establish *TaNRT1.1B* as a key regulator that links nitrogen nutrition to salt tolerance, offering a potential genetic target for improvement of stress resilience and nitrogen use efficiency in wheat.

## 4 Materials and methods

### 4.1 Plant materials and growth conditions

A panel of 13 hexaploid wheat (*Triticum aestivum*) varieties was used in this study: Jimai 60, Fielder, Qingmai 6, Cangmai 16, Cangmai 6005, Neixiang 188, Hengguan 35, Chinese Spring, Norin 67, Funo, Bainong 64, Jing 411, and Luyuan 502. All wheat varieties were grown in growth chambers under long-day conditions (16-h light/8-h dark) at 23 °C for phenotypic analyses and transformation experiments.

### 4.2 Plasmid construction and wheat transformation

To generate CRISPR/Cas9 constructs targeting *TaNRT1.1B*, two single-guide RNAs (sgRNAs) were designed to target the coding sequence of each homeolog and were inserted into the *pBUE413* vector as described previously [44].

Immature Fielder embryos were collected at 14 days post anthesis for *Agrobacterium*-mediated transformation using the EHA105 strain harboring the *TaNRT1.1B-pBUE413* construct as described previously [45].

To identify mutations in the target genes, gene-specific primers flanking the target sites were used to amplify genomic DNA fragments, and the PCR products were sequenced to confirm the mutations. Primers used for genotyping are listed in Table S4.

### 4.3 Salt treatment and phenotypic analysis of wheat seedlings

For hydroponic experiments, wheat seeds were germinated and grown in half-strength Hoagland solution. At the two-leaf-one-heart stage, uniformly sized seedlings were selected and exposed to 100 mM NaCl for various durations (0, 0.5, 3, 12, 24, 48, 72, and 96 h). Tissue nitrate content was measured as described previously [46].

In soil-culture experiments, wheat seedlings were grown to the one-leaf-one-heart stage and then treated with different concentrations of NaCl. The salt tolerance of Fielder and *tanrt1.1b* mutant seedlings was assessed after treatment with 150 mM NaCl for 14 days. Yield traits of Fielder and *tanrt1.1b* mutants were observed after treatment with 75 mM NaCl from the one-leaf-one-heart stage until the final harvest. The salt tolerance of different wheat genotypes was assessed after treatment with 100 mM NaCl for 21 days. For each cultivar, ten wheat seeds were sown per 1-gallon pot, and three pots were used as biological replicates. Plant height was recorded for each individual, and three plants with similar growth were selected and pooled for fresh weight measurement. The ratio of fresh weight under stressed versus control conditions was used as an indicator of salt tolerance.

### 4.4 Measurement of ROS-related physiological parameters

Histochemical staining with NBT and DAB was used to visualize levels of O_2_^−^ and H_2_O_2_, respectively. Antioxidant enzyme activities (SOD and POD) and ROS contents (H_2_O_2_ and O_2_^−^) were quantified using commercial assay kits according to the manufacturer's protocols (Solarbio, Beijing, China).

### 4.5 ABA treatment and phenotypic analysis of wheat seedlings

To investigate the effect of ABA on wheat growth, seeds were surface sterilized with 2% sodium hypochlorite for 15 min, washed five times with water, and then grown in germination pouches supplemented with 0 or 200 nM ABA for 4 days, after which root length and shoot height were recorded. Wheat seedlings grown hydroponically to the two-leaf-one-heart stage in half-strength Hoagland nutrient solution were treated with 0 or 200 nM ABA for 2 h, and the roots were harvested to examine the expression of ABA-responsive genes.

### 4.6 RNA-sequencing and data analysis

For bulk RNA-seq analysis, root and shoot tissues were collected from Jimai 60 seedlings treated with 100 mM NaCl for 0, 0.5, 3, 12, 24, or 48 h. Three biological replicates were used, each consisting of pooled tissues from six individual plants. Total RNA was extracted using TRIzol reagent (Invitrogen, Carlsbad, CA, USA), and RNA-seq was performed by Berry Genomics (Beijing, China). Raw reads were aligned to the wheat reference genome IWGSC RefSeq v2.0. Differential expression analysis was performed using the DESeq2 R package (v1.16.1). Genes with a fold change ≥ 2 and an adjusted *P*-value ≤ 0.05 were considered DEGs. GO enrichment analysis was performed using the GO.db R package (v3.20.0).

### 4.7 Clustering analyses

DEGs across time points were selected for clustering analysis using the Mfuzz R package (v2.66.0) [47]. Expression data were standardized using the standardize function to ensure a zero mean and unit variance for each gene. The optimal number of clusters was determined using the c selection function with the minimum centroid distance method, and a final clustering was performed with c = 6. Clusters with different temporal expression patterns were visualized using the mfuzz.plot2 function.

### 4.8 RNA extraction and RT–qPCR

Total RNA was extracted from frozen root and shoot tissues using the Ultrapure RNA Kit (CWBIO, Beijing, China) according to the manufacturer's instructions. First-strand cDNA was synthesized from 2 μg of total RNA using All-In-One 5X RT MasterMix with gDNA Removal (Applied Biological Materials, Vancouver, Canada). RT–qPCR was performed on an Applied Biosystems QuantStudio 3 instrument (Thermo Fisher Scientific, Waltham, MA, USA) using ChamQ Universal SYBR qPCR Master Mix (Vazyme, Nanjing, China). Gene-specific primers used for RT–qPCR are listed in Table S4. Relative expression levels were normalized to that of *TaACTIN* as an internal control and calculated using the 2^−ΔΔCt^ method. Three biological replicates were performed for each sample, with three technical replicates per biological replicate.

### 4.9 Statistical analyses

All statistical analyses were performed using GraphPad Prism (v8.0.1). Data are presented as means ± SD (*n* ≥ 3). Statistically significant differences were determined by Student's *t*-test or two-way analysis of variance (ANOVA) for multiple comparisons (∗∗∗∗*P* < 0.0001, ∗∗∗*P* < 0.001, ∗∗*P* < 0.01, ∗*P* < 0.05; ns, not significant).

### 4.10 Data and code availability

The raw RNA-seq data generated in this study have been deposited in the China National Center for Bioinformation (CNCB) Gene Expression Omnibus (GEO). All source data supporting the findings of this study are provided in the Supplementary Information. Sequences of genes used in this study are available at EnsemblPlants (https://plants.ensembl.org/index.html) under the following accession numbers: *TaNRT1.1B-A1*, TraesCS1A02G210900; *TaNRT1.1B-B1*, TraesCS1B02G224900; and *TaNRT1.1B-D1*, TraesCS1D02G214200. Custom scripts used for data analysis are available from the corresponding author upon reasonable request.

## CRediT authorship contribution statement

**Shuxuan Quan:** Writing – original draft, Investigation, Data curation, Methodology, Visualization, Funding acquisition. **Yitian Li:** Data curation, Validation. **Cheng Peng:** Investigation. **Qian Zhang:** Investigation. **Zhen Wang:** Visualization, Funding acquisition. **Jie Zang:** Data curation, Funding acquisition. **Guiluan Wang:** Data curation. **Xian Sheng Zhang:** Writing – review & editing, Funding acquisition, Conceptualization, Project administration. **Yifeng Hou:** Writing – review & editing, Conceptualization, Resources, Project administration, Methodology, Funding acquisition.

## Declaration of competing interest

The authors declare that they have no known competing financial interests or personal relationships that could have appeared to influence the work reported in this paper.

## Data Availability

The data that support the findings of this study are available from the corresponding author upon reasonable request.
