# Supplementary material for: TaNRT1.1B-mediated nitrogen acquisition contributes to salt adaptation and yield-trait maintenance in wheat
Source: aBIOTECH. 2026 Sep 12;7(4):100090. doi: 10.1016/j.abiote.2026.100090 (PMC13627928; doi:10.1016/j.abiote.2026.100090)
Supplement: Supplementary file 1 [file mmc2.docx]

**Fig. S1.** Effects of salt stress on shoot growth and expression of salt-responsive genes in wheat. **A** Morphological analysis of wheat shoots after treatment with 0 or 100 mM NaCl for the indicated time periods. Plants were grown hydroponically and treated at the two-leaf-one-heart stage. Scale bar = 1 cm. **B–C** Statistical analysis of shoot length (**B**) and shoot biomass (**C**) of the seedlings shown in (**A**). Data from 12 wheat seedlings per time point were used for statistical analysis. **D** Relative expression levels of the salt-responsive gene *TaSOS3* in roots under salt stress. Asterisks indicate statistically significant differences determined by Student’s *t*-test (*****P* < 0.0001, ***P* < 0.01, **P* < 0.05; ns, not significant).

**Fig. S2.** Transcriptional dynamics of wheat shoots under salt stress. **A** Principal component analysis (PCA) of RNA-seq data from wheat shoots treated with 100 mM NaCl for the indicated time periods. Each point represents a biological replicate, and colors indicate treatment duration. **B** Boxplots showing the distribution of FPKM values across all samples. The box represents the interquartile range, and the central line marks the median. The lower and upper hinges correspond to the first and third quartiles, respectively. **C** Numbers of upregulated and downregulated differentially expressed genes (DEGs) identified at each time point compared with the control (CK). DEGs were defined as genes with fold change ≥ 2 and adjusted *P* ≤ 0.05. **D** Venn diagram showing shared and unique DEGs identified in pairwise comparisons of different salt-treatment durations with the CK. **E** Volcano plot of DEGs identified in pairwise comparisons of different salt-treatment durations with the CK. Genes with adjusted *P* < 0.01 are highlighted in orange, and those with 0.01 ≤ adjusted *P* ≤ 0.05 are highlighted in blue.

**Fig. S3.** Transcriptional dynamics, clustering analysis, and functional annotation of salt-responsive genes in wheat shoots. **A** Heatmap showing the expression profiles of DEGs in shoots from different salt-treatment durations. Each row represents a gene, and each column represents a time point. Expression levels are *Z*-score normalized. **B** Fuzzy c-means clustering of DEGs in shoots using the Mfuzz R package. Each blue line depicts the expression pattern of one gene, and the cluster center for each group is shown in red. **C–H** GO enrichment analysis of DEGs in cluster 1 (**C**), cluster 2 (**D**), cluster 3 (**E**), cluster 4 (**F**), cluster 5 (**G**), and cluster 6 (**H**) as shown in (**B**). The top enriched biological process terms are shown for each cluster. In (**G**), terms related to nitrate assimilation and transport are highlighted in red.

**Fig. S4.** GO enrichment analysis of salt-responsive gene clusters in roots. **A–C** GO enrichment analysis of DEGs in cluster 2 (**A**), cluster 4 (**B**), and cluster 6 (**C**).

**Fig. S5.** Expression patterns of *NPF* and *NRT2* genes and FPKM values of *TaNRT1.1B* in wheat roots under salt stress. (Left) Heatmap showing the expression profiles of *NPF* and *NRT2* nitrate transporter genes in roots after treatment with 100 mM NaCl for the indicated time periods. Each row represents a gene, and each column represents a time point. Expression levels are *Z*-score normalized per gene (red, high expression; blue, low expression). (Right) FPKM values of *TaNRT1.1B* expression under salt stress.

**Fig. S6.** Transcriptional response of nitrate metabolism–related genes in wheat roots under salt stress. **A** Dot plot showing the expression patterns of key genes involved in nitrate transport and assimilation in roots across different salt-treatment durations. Abbreviations: NO_3_^−^, nitrate; NRTs, nitrate transporters; NRs, nitrate reductases; NO_2_^−^, nitrite; NiRs, nitrite reductases; NH_4_^+^, ammonium; GS/GOGAT, glutamine synthetase/glutamate synthase; Glu, glutamate. Expression levels are *Z*-score normalized per gene (red, high expression; blue, low expression). **B** RT–qPCR validation of selected nitrate metabolism–related genes identified from the RNA-seq data shown in (**A**). Expression levels were normalized to that of *TaACTIN* and calculated using the 2^−ΔΔCt^ method. Data are presented as means ± SD (*n* = 3 biological replicates). Asterisks indicate statistically significant differences compared with the control as determined by Student’s *t*-test (*****P* < 0.0001, ****P* < 0.001, ***P* < 0.01, **P* < 0.05, ns; not significant).

**Fig. S7.** Germination phenotypes of wild-type Fielder and *tanrt1.1b* mutants under salt stress. **A** Phenotypic analysis of germinating seeds of wild-type Fielder and *tanrt1.1b* mutants treated with 0 or 200 mM NaCl. Photographs were taken 72 h after treatment initiation. Scale bar = 1 cm. **B** Statistical analysis of germination rates of the seeds shown in (**A**). Data are presented as means ± SD (*n* = 3 biological replicates, each containing 16 seeds). Asterisks indicate statistically significant differences compared with the wild type as determined by Student’s *t*-test (*****P* < 0.0001).

**Fig. S8.** Relative expression of salt tolerance–related genes in wheat. **A–B** The relative expression of genes encoding antioxidant enzymes. **C–J** RT–qPCR analysis of salt tolerance–related genes in *TaNRT1.1B* knockout mutants (*tanrt1.1b-9* and *tanrt1.1b-18*). **K–P** Relative expression levels of genes encoding Na^+^ and K^+^ transporters in wheat. Data are presented as means ± SD (*n* = 3 biological replicates). Asterisks indicate statistically significant differences compared with the control as determined by Student’s *t*-test (****P* < 0.001, ***P* < 0.01, **P* < 0.05; ns, not significant).
